# Viewing Cute Images Does Not Affect Performance of Computerized Reaction Time Tasks

**DOI:** 10.3389/fpsyg.2021.800543

**Published:** 2022-01-14

**Authors:** Gal Ziv, Orly Fox

**Affiliations:** Motor Behavior Laboratory, The Academic College at Wingate, Netanya, Israel

**Keywords:** behavioral carefulness, cuteness, infantility, motor performance, reaction time

## Abstract

Humans are emotionally affected by cute or infantile appearances, typical of baby animals and humans, which in turn often leads to careful and cautious behavior. The purpose of this pre-registered study was to examine whether looking at cute images of baby pets improves performance of computerized cognitive-motor tasks. Ninety-eight participants were recruited for this online study and were randomly assigned to two experimental groups. The participants in one group performed two cognitive-motor tasks (Simon task and alternate task-switching task) before and after viewing images of adult pets and the participants in the other group performed the tasks before and after viewing images of baby pets. The participants who viewed images of baby pets rated them as significantly cuter (Cohen’s *d* = 0.50) and more infantile (Cohen’s *d* = 1.56) compared with those who viewed images of adult pets. All participants improved their performance from the pre-test to the post-test, but no differences in correct responses and reaction times were seen between the groups. However, pet ownership appeared to serve as a moderating variable with pet owners performing the Simon task faster than non-pet owners. In addition, pet owners reacted faster in the alternate task-switching task after viewing cute and infantile images but not after viewing images of adult pets. This effect was not found among non-pet owners. In conclusion, this study did not find that viewing cute images improves cognitive-motor performance, yet this may be dependent on moderators like pet ownership.

## Introduction

Cute or infantile physical appearances can elicit caregiving behaviors (Sherman et al., 2009), which are often performed in a careful manner, to avoid causing harm to the young. From an evolutionary perspective, such behavior makes sense, as it enables young to grow and thrive. Indeed, humans perceive children as more vulnerable, a perception that is related to emotions such as tenderness, sympathy, concern, and guilt about causing accidental harm (Dijker, 2010).

One question that arises from the emotional and behavioral responses to cute and infantile features, is whether the behavioral carefulness they elicit also leads to improved performance of cognitive-motor tasks. This question was examined in four studies, where participants were asked to look at images of either dogs/cats or puppies/kittens and perform a fine motor task (using tweezers to remove small pieces from holes without touching the edges of the holes; Sherman et al., 2009; Nittono et al., 2012, Exp. 1; Yoshikawa et al., 2020), a more complex task (Basketball free throwing; Yoshikawa and Masaki, 2021), or other cognitive/visual search tasks (Nittono et al., 2012, Exp. 2,3). In general, the results of those studies show that viewing images of baby pets leads to better and more accurate performance compared with viewing images of adult pets. For example, Nittono et al. (2012, Exp. 1) assigned 48 university students to two experimental groups. The participants performed a manual dexterity task before and after viewing images of either adult or baby pets. The participants who viewed images of baby pets improved their performance from the pre-test to the post-test by approximately 44%, but participants who viewed images of adult pets only improved by approximately 12%. In addition, the time required to complete the task was longer after viewing baby pets. However, in a second experiment (Nittono et al., 2012, Exp. 2), performance improvements were associated with faster task performance. In a more recent study (Álvarez-San Millán et al., 2021), viewing cute images led to longer reaction times (RT) in a global visual search task but shorter RTs in a local search task. Based on these studies it is not apparent whether the perception of cuteness leads to behavioral carefulness (and thus longer performance durations) or to improved attention (and thus shorter performance durations).

Another study (Yoshikawa et al., 2020) examined the Quiet Eye (QE) duration in addition to performance. The QE is the final fixation on a specific location in the visuomotor space that begins before a critical movement of a motor task (Vickers, 2016). Longer QE durations have been associated with expert and successful performance (for a review, see Lebeau et al., 2016). Yoshikawa et al. (2020) assigned 28 university students to two groups. The participants performed a manual dexterity task before and after viewing images of baby or adult pets. The participants who viewed images of baby pets perceived them as cuter and more infantile, and presented improved performance and increased QE durations in the post-test compared with the pre-test. Improved performance after viewing cute images was also reported in a pressure test that followed the post-test. However, there was no improved performance or increased QE durations among the participants who viewed adult pets.

The abovementioned studies showed positive associations between viewing cute images and performance but represent a small body of literature. In addition, these studies utilized a variety of motor and cognitive tasks that may be more or less sensitive to an intervention that attempts to elicit behavioral carefulness. In the current study, we chose two tasks that require attention—a Simon task and an alternate task switching task. In a Simon task, participants are required to attend to possible incongruence between the meaning of a cue and its location (e.g., Lu and Proctor, 1995). In an alternate task switching task, participants must attend to the rule change when there is a switch between two (or more) tasks, each requires attention to a different attribute of a stimulus (Monsell, 2003). We chose these tasks for three main reasons. First, if perceived cuteness or infantility leads to behavioral carefulness, then attention should be affected because it is required for careful or mindful behavior. Second, these tasks require both accuracy and speed and are therefore suitable for assessing whether participants adopt behavioral carefulness because it should affect the speed-accuracy tradeoff in performance. Finally, these tasks are suitable for online studies because they are relatively simple and can be completed in a short duration. In addition, these tasks have been shown to be sensitive enough to show effects of other cognitive interventions in online studies (e.g., Ziv and Lidor, 2021).

Therefore, the purpose of this study was to attempt to replicate previous findings on perceived cuteness and behavioral carefulness. For this purpose, we developed an online study that utilized computerized RT tasks. We chose to use an online research methodology with covert participant recruitment, to ensure double-blinding and to allow us to reach an adequate sample size for achieving ample statistical power. We hypothesized that: (1) in both a Simon task and an alternate task-switching task, participants who viewed images of puppies/kittens would exhibit more correct responses compared with participants who viewed images of adult pets; and (2) in both a Simon task and an alternate task-switching task, participants who viewed images of puppies/kittens would react slower than participants who viewed images of adult pets. Both hypotheses are based on the concept of behavioral carefulness, although, at least for the second hypothesis, there is data to support either longer performance durations (careful behavior) or shorter performance durations (improved attention).

## Methods

### Pre-registration and Raw Data Repository

The study was pre-registered on aspredicted.org.^1^ Analyses that were not pre-registered are reported as ‘‘exploratory analyses.’’ The raw dataset is available on OSF.^2^

### Preliminary Image Selection

We showed 20 undergraduate students (12 females) 15 images of adult dogs/cats and 15 images of puppies/kittens. These images were taken from an Internet depository of freely useable images.^3^ The participants were asked to rate each image on a scale of 0 (not at all) to 5 (very much) according to five attributes: cuteness, excitement, pleasantness, infantility, and the desire to get closer to the animal. There were no differences in ratings between males and females (all *p*-values > 0.20) and the ratings are presented in Table 1. Based on these ratings, we chose for the main study seven images of puppies/kittens and seven images of adult dogs/cats that had the highest and the lowest cuteness and infantility ratings, respectively, while attempting to minimize differences in pleasantness, excitement, and the desire to get closer to the animal.

**TABLE 1:** Ratings of the seven images of adult dogs/cats and the seven images of puppies/kittens that were chosen for the main study.

Characteristic	Adult dogs/cats	Puppies/kittens	Statistics
Cuteness	2.3 ± 1.1	4.4 ± 0.6	*t*(19) = 9.7, *p* < 0.01
Infantility	1.3 ± 1.2	3.9 ± 1.3	*t*(19) = 8.3, *p* < 0.01
Pleasantness	2.6 ± 1.1	4.2 ± 0.7	*t*(19) = 6.9, *p* < 0.01
Excitability	2.1 ± 1.2	3.7 ± 1.1	*t*(19) = 7.9, *p* < 0.01
Wanting to get closer	2.4 ± 1.3	4.1 ± 0.9	*t*(19) = 6.6, *p* < 0.01

### Main Study

#### Participants

We used G*Power (Faul et al., 2007) to perform an *a priori* power analysis for a two-way analysis of variance (ANOVA) [Group (baby/adult pets’ images) × Test (pre/post)]. Previous studies on this topic found moderate to large effect sizes (e.g., Cohen’s d ∼ 0.6; Sherman et al., 2009, η^2^*_*p*_* = 0.17–0.19; Nittono et al., 2012, η^2^*_*p*_* = 0.12–0.15; Yoshikawa et al., 2020, η^2^*_*p*_* = 0.11–0.23; Yoshikawa and Masaki, 2021). Therefore, we selected a moderate effect size (Cohen’s *d* = 0.5/Cohen’s *f* = 0.25) and the following parameters: alpha (two-sided) = 0.05, power = 0.80, and a correlation of 0.5 among the repeated measures. The results of the power analysis suggested that 98 participants were required to detect differences between groups with 80% power, and to find differences within groups or an interaction with > 99% power. To do so, we recruited 100 participants through Prolific^4^ —an online participant database platform that allows participants to participate in online studies from their own computer. (Two participants were removed from the study—see Data Exclusion section).

The 98 participants were randomly allocated to two groups: (1) puppies/kittens’ group (*n* = 48, 27 females, 2 did not report gender, mean age = 23.9 ± 4.2 years); and (2) adult dogs/cats’ group (*n* = 50, 31 females, mean age = 23.4 ± 3.9 years). Randomization to groups was automatically performed by the web-based platform by randomly allocating three of every six participants to each group. Group imbalance occurred because with such online studies, participants can decide to withdraw at any time, without the researcher’s knowledge—a situation that the automatic randomization process cannot always account for. The participants were paid £ 2.5 for their participation. The study was approved by the ethics committee of the Academic College at Wingate (approval # 313), and all participants signed an informed consent form (at the preliminary image selection stage) or completed an electronic informed consent form on the study’s website prior to participation (for the main study).

#### Tasks

##### Simon Task

The words “right” or “left” were displayed on the right or left side of a centralized cross. The participants were required to press the “j” key if they saw the word “right” (even if it appeared to the left of the cross) and to press the “f” key if they saw the word “left” (even if it appeared to the right of the cross) (Simon and Wolf, 1963; Lu and Proctor, 1995). The words “right” or “left” were presented for 900 ms, followed by 600 ms during which only the centralized cross was displayed. The next word was presented after 600 ms passed whether the participants pressed a key or not.

##### Alternate Task-Switching Task

A square or rectangle, in either blue or green, appeared at the top or at the bottom of the screen. If the shape appeared at the top of the screen, the participants were asked to press the “f” key if the shape was blue and the “j” key if the shape was green (regardless of the type of shape); If the shape appeared at the bottom of the screen, the participants were asked to press “f” if the shape was a square, and “j” if it was a rectangle (regardless of the color). In this task, each stimulus was presented for an unlimited duration until a pressing of key was recorded. The task included the presentation of two shapes at the top of the screen or two shapes at the bottom of the screen, alternately.

#### Procedure

This study was conducted online using a web-based platform^5^ (Anwyl-Irvine et al., 2020). Web-based studies have been shown to provide accurate measures of RT that are similar to those attained in lab-based studies (e.g., Crump et al., 2013; Hilbig, 2016). Participation was allowed from either a desktop or a laptop computer only and it was not possible to participate using a tablet or a smartphone.

The participants performed eight familiarization trials for both types of tasks, followed by a pre-test that included three blocks of 24 trials for each of the two tasks. Then, based on the group to which they were assigned, the participants were asked to rate seven images of either adult or baby pets from their most favorite to the least favorite. The participants watched each image separately in random order and then saw a screen that showed all seven images and a text box in which they entered the numbers of their preferred images in order. The participants then performed a post-test that included an additional three blocks of 24 trials for both tasks. In both the pre-test and the post-test, the participants were instructed to perform the tasks as fast and as accurately as possible. After completing the post-test, the participants were asked to rate each of the seven pets based on five qualities: cuteness, excitement, pleasantness, infantility, and wanting to get closer, on a scale of 0 (not at all) to 5 (very much). Finally, the participants were asked to rate their love of dogs and their love of cats on a scale of 0 (not at all) to 5 (very much), and whether they currently own a pet. Tasks were performed in a counterbalanced order during the familiarization stage and during the pre- and post-tests.

#### Data Exclusion

In the Simon task, we excluded RT values of over 1,000 ms and blocks with 50% errors or more. This led to the removal of eight blocks in the pre-test and seven blocks in the post-test. In the alternate task-switching task, we excluded RT values of over 2,500 ms and blocks with 50% errors or more. This led to the removal of 28 blocks from the pre-test and 21 blocks from the post-test. Three of those blocks were removed from the data of one participant with RT values of approximately 100 ms, which are impossible in these tasks. Two participants were removed from the study because they had more than two blocks that had to be removed from one of the two tasks.

#### Data Analysis

We measured RTs (ms) and the number of correct responses. These measures were averaged for the three blocks of trials in both the pre-test and the post-test. We used RT values for all trials because we wanted to separate speed of response from accuracy of response and because there were no differences between mean RTs when all trials were averaged or when only correct trials were averaged (all *p*-values > 0.05, differences < 3 ms). Based on skewness and kurtosis values, in most cases the RTs and number of correct responses were normally distributed and were analyzed using a two-way ANOVA [Group (adult/baby pets’ images) × Test (pre/post)]. The number of correct key presses in the Simon task in both the pre- and post-tests was not normally distributed, and we used the Mann-Whitney test to separately examine differences between the two groups in the pre-test and in the post-test. We also conducted a Bayesian analysis to assess the probability of null findings compared with significant findings. For this purpose, we report BF_01_ values that show the probability of the null (or tested) hypothesis compared with alternative hypotheses. Bonferroni *post hoc* analyses and 95% confidence intervals were used for *post hoc* testing when appropriate, and alpha was set at 0.05. In cases where the *p*-value was greater than 0.05 but less than 0.10, and the effect size was moderate or higher (Cohen’s *d* ≥ 0.5 or η^2^*_*p*_* ≥ 0.06), we considered this finding as practically relevant and discuss it as such. Statistical analyses were conducted using SPSS version 25 (SPSS Statistics, IBM, United States) and JASP (JASP Team, 2021) for the Bayesian analyses.

## Results

Table 2 presents the differences between the two groups in RTs and correct responses for both the Simon and the alternate task switching tasks during the pre-test and the post-test, as well as the assessment of the images (with differences only being found in *cuteness* and *infantility*).

**TABLE 2:** Reaction times, correct responses, and ratings of the seven images of adult dogs/cats and the seven images of puppies/kittens of the participants in the main study.

Task	Variable	Test	Adult dogs/cats	Puppies/kittens	Statistics
Simon task	Reaction time (ms)	Pre-test	532.98 ± 63.77	549.76 ± 66.66	See text
		Post-test	521.12 ± 65.98	533.87 ± 71.48	
	Correct responses	Pre-test	21.91 ± 2.43	22.29 ± 2.13	See text
		Post-test	22.18 ± 2.48	22.42 ± 2.52	
Alternate task switching task	Reaction time (ms)	Pre-test	1,255.58 ± 534.11	1,232.91 ± 511.00	See text
		Post-test	1,065.94 ± 379.73	999.36 ± 316.66	
	Correct responses	Pre-test	20.37 ± 3.96	21.02 ± 3.19	See text
		Post-test	21.75 ± 2.53	22.08 ± 2.72	
Rating images	Cuteness		3.8 ± 0.6	4.1 ± 0.6	*t*(96) = 2.3, *p* = 0.03
	Infantility		2.2 ± 0.9	3.6 ± 0.9	*t*(96) = 7.7, *p* < 0.01
	Pleasantness		3.5 ± 0.8	3.7 ± 0.6	*t*(95) = 1.7, *p* = 0.10
	Excitability		2.8 ± 1.0	2.9 ± 0.9	*t*(96) = 0.1, *p* = 0.96
	Wanting to get closer		3.2 ± 1.0	3.5 ± 0.8	*t*(96) = 1.6, *p* = 0.12

*Mean ± SD.*

### Time Spent Rating Preferred Images

There were no differences between groups in the time spent looking at the pet images, *t*(96) = 1.39, *p* = 0.17, Cohen’s *d* = 0.28. The participants who watched baby pets and the participants who watched adult pets spent 34.30 ± 27.98 s and 27.27 ± 21.86 s, respectively, watching the seven images. There were also no differences between groups in the time spent rating the preferred images, *t*(96) = 0.56, *p* = 0.58, Cohen’s *d* = 0.11. The participants who watched baby pets and the participants who watched adult pets spent 52.22 ± 24.83 sand 49.48 ± 23.86 s, respectively, rating the images based on their preferences.

### Differences Between Males and Females

The only difference between males and females in images’ ratings was in the pleasantness attribute, *t*(93) = 2.14, *p* = 0.035, Cohen’s *d* = 0.46 (for all other attributes, *p*-values > 0.11). However, this difference disappeared after using a False Discovery Rate procedure (Benjamini and Hochberg, 1995) to account for multiple comparisons. There were also no gender differences in RTs and correct responses for both the Simon task and the alternate task switching task (all *p*-values > 0.10).

### Simon Task

#### Reaction Times

A two-way ANOVA [Group × Test] with repeated measures on the Test factor revealed a Test effect, *F*(1, 95) = 8.77, *p* = 0.004, η^2^*_*p*_* = 0.09. The mean RT was 536.90 ± 63.22 ms in the pre-test and 524.53 ± 65.71 ms in the post-test. There was no group effect, *F*(1, 95) = 1.85, *p* = 0.18, η^2^*_*p*_* = 0.02, and no interaction, *F*(1, 95) = 0.04, *p* = 0.85, η^2^*_*p*_* = 0.00.

#### Correct Responses

There were no differences between groups in the pre-test (Mann-Whitney *U* = 1,002.50, *p* = 0.21; mean: 22.44 ± 1.39 out of 24 trials) or in the post-test (Mann-Whitney *U* = 969.00, *p* = 0.10; mean: 22.64 ± 1.22 out of 24 trials).

### Alternate Task-Switching Task

#### Reaction Times

A two-way ANOVA [Group × Test] with repeated measures on the Test factor revealed a Test effect, *F*(1, 89) = 47.74, *p* < 0.001, η^2^*_*p*_* = 0.35. The mean RT was 1,175.87 ± 350.22 ms in the pre-test and 999.10 ± 279.22 ms in the post-test. There was no group effect, *F*(1, 89) = 0.21, *p* = 0.64, η^2^*_*p*_* = 0.00, and no interaction, *F*(1, 89) = 0.13, *p* = 0.72, η^2^*_*p*_* = 0.00.

#### Correct Responses

A two-way ANOVA [Group × Test] with repeated measures on the Test factor revealed a Test effect, *F*(1, 89) = 19.72, *p* < 0.001, η^2^*_*p*_* = 0.18. The mean correct responses were 21.34 ± 2.75 in the pre-test and 22.35 ± 1.85 in the post-test (out of 24 trials). There was no group effect, *F*(1, 89) = 0.09, *p* = 0.92, η^2^*_*p*_* = 0.00, and no interaction, *F*(1, 89) = 0.75, *p* = 0.39, η^2^*_*p*_* = 0.01.

### Bayesian Analysis

We used a two-way Bayesian ANOVA to assess the probability of all models compared to the best model. For the Simon RT, the best model only included the Test factor. BF_01_ for the Group factor, the Group and Test factor, and the interaction was 11.34, 1.48, and 2.77, respectively. For the alternate task-switching task, the best model only included the Test factor. BF_01_ for the Group factor, the Group and Test factor, and the interaction was 4.25e7, 2.84, and 3.89, respectively. For the correct response of the alternate task-switching task, the best model only included the Test factor. BF_01_ for the Group factor, the Group and Test factor, and the interaction was 2,599.14, 3.36, and 3.62, respectively. These Bayes Factors mostly provide moderate to strong evidence that models that include the Group factor or the interaction are less likely than the model that includes the Test factor alone. This is based on the following interpretation: 3 < BF < 10 can be considered moderate evidence and BF > 10 can be considered strong evidence (van Doorn et al., 2021).

## Exploratory Analysis

### Pet Ownership and Images Ratings

We conducted a two-way ANOVA (Group × Pet ownership) on each of the five attributes that were rated for each image. There were 49 pet owners and 47 non-pet owners (two participants did not report pet ownership and were not included in the analysis).

#### Cuteness

**FIGURE 1:**
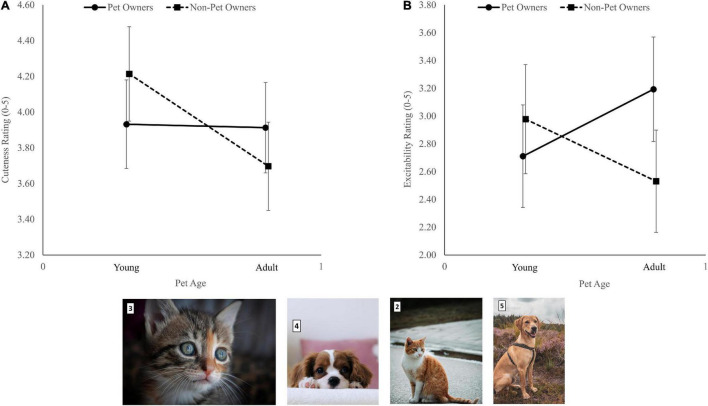
The interaction between pet ownership and ratings of young and old pets’ images for cuteness **(A)** and excitability **(B)**. Error bars represent 95% confidence intervals. Photos are samples of the baby (3 and 4) and adult (2 and 5) pets’ photos used in this study (Photo # 2 by Daniel Mačura on Unsplash; photo # 3 by Adél Gröber on Unsplash; photo # 4 by T.R Photography on Unsplash; Photo # 5 by Sam te Kiefte on Unsplash).

There was a Group effect, *F*(1, 92) = 4.42, *p* = 0.04, η^2^*_*p*_* = 0.05 (values in Table 2). There was no Pet ownership effect, *F*(1, 92) = 0.07, *p* = 0.79, η^2^*_*p*_* = 0.00, and no interaction, *F*(1, 89) = 3.81, *p* = 0.054, η^2^*_*p*_* = 0.04. However, an examination of the 95% confidence intervals (CI) revealed that while pet owners rated images of baby and adult pets similarly (∼3.90), non-pet owners rated baby pets higher than adult pets [4.21 (CI 3.95-4.48) vs. 3.70 (CI 3.45-3.95), respectively, Figure 1A].

#### Infantility

There was a Group effect, *F*(1, 92) = 57.39, *p* < 0.001, η^2^*_*p*_* = 0.38 (values in Table 2). There was no Pet ownership effect, *F*(1, 92) = 0.72, *p* = 0.40, η^2^*_*p*_* = 0.01 and no interaction, *F*(1, 92) = 1.87, *p* = 0.174, η^2^*_*p*_* = 0.02.

#### Pleasantness

There was no Group effect, *F*(1, 91) = 2.35, *p* = 0.13, η^2^*_*p*_* = 0.03, no Pet ownership effect, *F*(1, 91) = 0.01, *p* = 0.92, η^2^*_*p*_* = 0.00, and no interaction, *F*(1, 91) = 0.57, *p* = 0.45, η^2^*_*p*_* = 0.01.

#### Excitability

There was a significant Group × Pet ownership interaction, *F*(1, 92) = 6.00, *p* = 0.02, η^2^*_*p*_* = 0.06. While pet owners rated adult pets as more exciting than baby pets, non-pet owners rated baby pets as more exciting than adult pets (Figure 1B). There was no Group effect, *F*(1, 92) = 0.01, *p* = 0.93, η^2^*_*p*_* = 0.00, and no Pet ownership effect, *F*(1, 91) = 1.08, *p* = 0.30, η^2^*_*p*_* = 0.01.

#### Wanting to Get Closer

There was no Group effect, *F*(1, 92) = 1.88, *p* = 0.17, η^2^*_*p*_* = 0.02, no Pet ownership effect, *F*(1, 92) = 1.68, *p* = 0.20, η^2^*_*p*_* = 0.02, and no interaction, *F*(1, 92) = 0.12, *p* = 0.73, η^2^*_*p*_* = 0.00.

### Pet Ownership and Performance

Because we found that pet ownership can affect how individuals assess images of baby and adult pets, we reanalyzed the performance data with a three-way ANOVA [Group (adult/baby pets’ images) × Pet ownership (owner/non-owner) × Test (pre/post)] with repeated measures on the Test factor. The only variable that was not reanalyzed was the correct responses in the Simon task because it did not present a normal distribution.

#### Simon Task Reaction Times

The analysis revealed a Pet ownership effect, *F*(1, 91) = 4.08, *p* = 0.046, η^2^*_*p*_* = 0.04. Pet owners had faster RTs (518.98 ± 65.31 ms) compared with non-pet owners (543.51 ± 55.80 ms). Similar to the pre-registered two-way ANOVA, there was also a Test effect, *F*(1, 91) = 9.25, *p* = 0.003, η^2^*_*p*_* = 0.09. There was no Group effect, *F*(1, 91) = 2.03, *p* = 0.16, η^2^*_*p*_* = 0.02, no Group × Pet ownership interaction, *F*(1, 91) = 0.06, *p* = 0.81, η^2^*_*p*_* = 0.00, no Test × Pet ownership interaction, *F*(1, 91) = 0.32, *p* = 0.57, η^2^*_*p*_* = 0.00, no Test × Group interaction, *F*(1, 91) = 0.19, *p* = 0.67, η^2^*_*p*_* = 0.00, and no three-way interaction, *F*(1, 91) = 0.004, *p* = 0.95, η^2^*_*p*_* = 0.00.

#### Alternate Task-Switching Task Reaction Times

**FIGURE 2:**
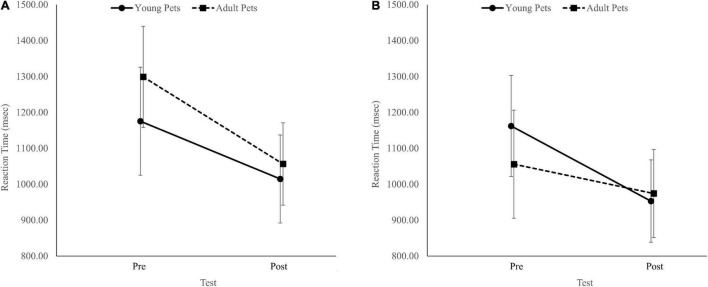
The interaction between groups (adult pets’ images/young pets’ images) and test timing (pre/post) for non-pet owners **(A)** and pet owners **(B)** for the alternate task-switching task reaction time. Error bars represent 95% confidence intervals.

The analysis revealed a Group × Pet ownership × Test interaction, *F*(1, 86) = 4.24, *p* = 0.04, η^2^*_*p*_* = 0.05. In non-pet owners, RTs improved similarly in both groups from the pre-test to the post-test (Figure 2A). However, among pet owners, RTs only improved in the group that looked at baby pets (Figure 2B). Similar to the pre-registered two-way ANOVAs, there was also a Test effect, *F*(1, 86) = 46.60, *p* < 0.001, η^2^*_*p*_* = 0.35. There was no Group effect, *F*(1, 86) = 0.11, *p* = 0.75, η^2^*_*p*_* = 0.00, no Pet ownership effect, *F*(1, 86) = 2.62, *p* = 0.11, η^2^*_*p*_* = 0.03, no Group × Pet ownership interaction, *F*(1, 86) = 1.03, *p* = 0.31, η^2^*_*p*_* = 0.01, no Test × Group interaction, *F*(1, 86) = 0.20, *p* = 0.65, η^2^*_*p*_* = 0.00, and no Test × Pet ownership interaction, *F*(1, 86) = 1.22, *p* = 0.27, η^2^*_*p*_* = 0.01.

#### Alternate Task-Switching Task Correct Responses

Similar to the pre-registered two-way ANOVA, there was a Test effect, *F*(1, 86) = 20.71, *p* < 0.001, η^2^*_*p*_* = 0.19. There was no Group effect, *F*(1, 86) = 0.06, *p* = 0.81, η^2^*_*p*_* = 0.00, no Pet ownership effect, *F*(1, 86) = 1.39, *p* = 0.24, η^2^*_*p*_* = 0.02, no Group × pet ownership interaction, *F*(1, 86) = 0.84, *p* = 0.36, η^2^*_*p*_* = 0.01, no Test × Group interaction, *F*(1, 86) = 1.09, *p* = 0.30, η^2^*_*p*_* = 0.01, no Test × Pet ownership interaction, *F*(1, 86) = 3.15, *p* = 0.08, η^2^*_*p*_* = 0.04, and no three-way interaction, *F*(1, 91) = 0.70, *p* = 0.40, η^2^*_*p*_* = 0.01.

### Comparison of Top 20% vs. Bottom 20% of Cuteness Ratings

In the current study, the differences in cuteness ratings of adult and baby pets were significant but with an effect size smaller than reported in previous studies (for comparison of ratings of cuteness and infantility in previous studies and in the current study, see Table 3). Therefore, we examined the differences in performance between participants in the top 20% of cuteness rating (mean = 4.8 ± 0.2) and those in the bottom 20% of this rating (mean = 3.0 ± 0.3). This was accompanied by a difference in the rating if infantility (3.6 ± 1.0 for the top 20% and 2.1 ± 1.2 for the bottom 20%). These values are similar to those seen in previous studies. The results of this analysis were similar to our main analysis with an improvement from pre- to post-test, but no other significant effects or interactions. BF_01_ of the Group × Test interaction for the Simon RT was 1.24, and for the alternate task-switching task RT and correct responses, 0.97 and 2.56, respectively.

**TABLE 3:** Comparison of ratings of cuteness and infantility in previous studies and in the current study (on a 6-point scale, either 0–5 or 1–6).

Attribute	Study	Adult dogs/cats	Puppies/kittens
Cuteness	Sherman et al., 2009, Exp. 1	3.2 ± 0.3	4.8 ± 0.1
	Sherman et al., 2009, Exp. 2	3.1 ± 0.3	4.3 ± 0.2
	Nittono et al., 2012, Exp. 1	4.2 ± 0.8	4.8 ± 0.6
	Nittono et al., 2012, Exp. 2	3.8 ± 0.4	5.0 ± 0.3
	Nittono et al., 2012, Exp. 3	3.9 ± 0.6	5.0 ± 0.7
	Yoshikawa et al., 2020	3.9 ± 0.3	4.7 ± 0.2
	Yoshikawa and Masaki, 2021, males	3.7 ± 0.3	4.9 ± 0.2
	Yoshikawa and Masaki, 2021, females	4.2 ± 0.2	4.5 ± 0.3
	Current study	3.8 ± 0.6	4.1 ± 0.6
Infantility	Sherman et al., 2009, Exp. 1	NA	NA
	Sherman et al., 2009, Exp. 2	NA	NA
	Nittono et al., 2012, Exp. 1	2.7 ± 0.6	4.8 ± 0.7
	Nittono et al., 2012, Exp. 2	2.3 ± 0.7	5.0 ± 0.4
	Nittono et al., 2012, Exp. 3	2.5 ± 0.7	5.0 ± 0.6
	Yoshikawa et al., 2020	2.8 ± 0.2	4.5 ± 0.2
	Yoshikawa and Masaki, 2021, males	1.8 ± 0.2	5.6 ± 0.1
	Yoshikawa and Masaki, 2021, females	1.9 ± 0.2	4.9 ± 0.2
	Current study	2.2 ± 0.9	3.6 ± 0.9

### Analyses of Switch vs. No-Switch Trials

We performed this analysis to assess whether differences between groups were related to either the switch trials (the trial after rule change) or the no-switch trials (the trial when the rule remains the same). For these analyses, we excluded five participants who consistently had six correct responses or lower in both switch and no-switch trials. For RT, a 3-way ANOVA [Group (adult/baby pets’ images) × Switch (yes/no) × Test (pre/post)] revealed a Switch effect, *F*(1, 92) = 194.15, *p* < 0.001, η^2^*_*p*_* = 0.68. The RT in the switch trials (1,305.45 ± 468.04 ms) was slower than the RT in the no-switch trials (977.46 ± 378.61 ms). There was also a Test effect, *F*(1, 92) = 41.10, *p* < 0.001, η^2^*_*p*_* = 0.31. The RT in the pre-test (1,257.08 ± 527.53 ms) was slower than the RT in the post-test (1,025.80 ± 348.59 ms). There were no other main effects or interactions (all *p*-values > 0.05).

For correct responses, the 3-way ANOVA revealed a three-way interaction, *F*(1, 92) = 4.71, *p* = 0.03, η^2^*_*p*_* = 0.05, a Test effect, *F*(1, 92) = 15.23, *p* < 0.001, η^2^*_*p*_* = 0.14, and a Switch effect, *F*(1, 92) = 6.05, *p* = 0.02, η^2^*_*p*_* = 0.06. However, these effects had no practical significance as all average correct response values ranged between 10.56 and 11.24 and even significant differences were within the 95% confidence intervals of each other. There were no other main effects or interactions (all *p*-values > 0.05).

## Discussion

The purpose of this study was to examine whether looking at images of puppies/kittens would lead to improved performance in computerized cognitive-motor tasks. We hypothesized that participants who viewed images of puppies/kittens would have more correct responses and slower RTs compared with participants who viewed images of adult dogs/cats. Our data, however, did not support our hypotheses. In addition, Bayes analysis provided moderate to strong evidence that performance improvements from the pre-test to the post-test are more likely than group differences or interactions, and inconclusive evidence for a combination of the Test and Group factors. Our null findings could be explained by the small difference in the rating of cuteness between our two experimental groups compared with previous studies (see Table 3). However, these findings persisted even when comparing only the top 20% and the bottom 20% of cuteness ratings. In this comparison, differences in ratings of cuteness and infantility were similar to those obtained in previous studies (Sherman et al., 2009; Nittono et al., 2012; Yoshikawa et al., 2020; Yoshikawa and Masaki, 2021). Another possible explanation for the lack of significant findings in the current study is the tasks that we used. Previous studies used fine motor tasks (e.g., Sherman et al., 2009; Nittono et al., 2012; Yoshikawa et al., 2020). In the current study, the tasks required cognitive inhibition or task switching, as well as fast responses but did not require dexterity. It is possible that behavioral carefulness that follows viewing cute babylike images mainly affects fine motor skills. However, a recent study (Yoshikawa and Masaki, 2021), found that viewing cute images leads to improved free-throwing performance in a no-pressure test—a skill that requires accuracy but is not considered a fine motor task.

Unlike previous studies, at the end of the current study we asked participants whether they currently own a pet and in an exploratory analysis we examined whether pet ownership may moderate the results. This analysis showed that: (1) pet owners rated some attributes of adult/baby pets differently than non-pet owners; (2) pet owners performed faster than non-pet owners in the Simon task; and (3) pet-owners improved their RTs from the pre-test to the post-test in the alternate task-switching task, but only when looking at images of baby pets, not adult pets. This interaction was not found among non-pet owners. These findings suggest that pet ownership can moderate the relationship between perceiving cuteness/infantility and cognitive-motor performance. While it is not apparently clear why pet owners would outperform non-pet owners, it is possible that pet owners experienced a higher positive affect when looking at images of pets. Indeed, it has been reported that a positive affect can influence the performance of various cognitive tasks (Ashby et al., 1999). It is also possible that pet owners experienced a higher positive affect when viewing images of baby pets compared with adult pets, which may have led to the interaction in the alternate task-switching task. This should be directly examined in future studies. Finally, we hypothesized that viewing cute images would lead to longer RTs due to behavioral carefulness, yet our findings show that with pet-owners, the opposite occurred. It is possible, as Sherman et al. (2009) suggested, that perceived cuteness increased attention and motor control, rendering shorter RTs after viewing cute images. In addition, this finding is in line with Álvarez-San Millán et al. (2021) who found shorter RTs in a local (but not global) search task, and with Nittono et al. (2012, Exp. 2) who reported faster task performance in a visual matrices search.

The fact that the moderating effect of pet ownership differed between tasks suggests that the effects of behavioral carefulness on task performance may be task dependent. It is possible, for example, that the Simon task was too simple to reveal an effect (see section “Strengths and Limitations of the Current Study”) while the difficulty of the alternate task switching task was enough to expose the effect. Task difficulty has been previously shown to moderate the effects of different learning interventions and it seems that such effects are more pronounced in more difficult task conditions (e.g., attentional focus: Wulf et al., 2007; Feedback: Abbas and North, 2018).

In the current study, gender did not have the same effect as pet ownership. Gender differences did not lead to differences in ratings of any of the five attributes of baby or adult pets in both the preliminary image selection stage and the main experiment. Yoshikawa and Masaki (2021) reported contrasting gender differences (i.e., no gender differences in the ratings of cuteness, pleasantness, and excitement; but males rated baby animals higher than females on infantility and females rated baby animals higher than males on approach motivation). In other previous studies, compared with males, females were more sensitive to differences in cuteness between human babies (e.g., Sprengelmeyer et al., 2009; Lobmaier et al., 2010). In addition, Lehmann et al. (2013) showed that females were more sensitive than males to the human and animal baby schema effect—the adults’ innate mechanisms to protect and nurture the young (Lorenz, 1943, as cited in Lehmann et al., 2013). We suggest that gender differences deserve further investigation. Rating cuteness may not be necessarily the same as sensitivity to the baby schema effect. It is possible, for example that females rate animal cuteness similarly to males while at the same time respond to the baby schema effect more strongly than males. Thus, images of adult and baby humans (rather than pets) could provide more accurate input and may be more suitable to assess behavioral carefulness in humans.

### Strengths and Limitations of the Current Study

One strength of the current study is that our sample size provided us with ample statistical power. Moreover, by using an online study format, we ensured double blinding as the researchers had no contact whatsoever with the participants, who received the instructions and performed the tasks on their personal computer.

One limitation of this online study, however, is that it is difficult to know whether the participants followed the exact instructions. Therefore, we removed data from blocks with 50% errors or higher, as in both tasks, participants could simply have repeatedly pressed one single key (i.e., “f” or “j”) time and again, and achieve 50% correct responses without reading or following the instructions. Another possible imitation is that the scale of the task may have differed on participants’ screens. However, we do not expect this to affect the results because we limited participation to laptops and desktops (but not tablets or smartphones) and therefore, even the smallest computer screen should have provided a large enough view of the tasks. In addition, in an online study it is difficult to know whether participants engage with the tasks as instructed and therefore, results should be interpreted with caution. However, even though we had little control over the environment, the equipment the participants used, or their motivation to perform the task as instructed, large sample sizes that are more easily attained in online studies can make up for such shortcomings (Woods et al., 2015). A third limitation is the use of images of both dogs and cats. Using images of just one species would eliminate differences due to participants’ disliking only dogs or only cats. It is also possible that the null findings in correct responses were due to a ceiling effect because participants showed over 89 and 94% accuracy in the alternate task switching task and the Simon task, respectively.

In conclusion, the current online study did not replicate previous findings whereby viewing cute images leads to improved performance. However, pet ownership is a possible moderator of the relationship between participants’ reactions to cute images of baby and adult pets and their subsequent performance.

## Data Availability Statement

The datasets presented in this study can be found in online repositories. The names of the repository/repositories and accession number(s) can be found below: OSF (https://osf.io/g3j69/?view_only=fe23edab18bc40808b1f274b9ceeea15).

## Ethics Statement

The studies involving human participants were reviewed and approved by the Ethics Committee of the Academic College at Wingate. Written informed consent was not provided because the main study was an online study. Participants filled an electronic informed consent as was approved by the ethics committee and as written in the manuscript. All participants in the preliminary image selection stage signed an informed consent form as this part was conducted in person.

## Author Contributions

GZ: conceptualization, methodology and data collection, formal analysis, and writing—original draft. OF: conceptualization, methodology and data collection, and writing—reviewing and editing. Both authors contributed to the article and approved the submitted version.

## Conflict of Interest

The authors declare that the research was conducted in the absence of any commercial or financial relationships that could be construed as a potential conflict of interest.

## Publisher’s Note

All claims expressed in this article are solely those of the authors and do not necessarily represent those of their affiliated organizations, or those of the publisher, the editors and the reviewers. Any product that may be evaluated in this article, or claim that may be made by its manufacturer, is not guaranteed or endorsed by the publisher.

## Footnotes

1
https://aspredicted.org/zq3zb.pdf

2
https://osf.io/g3j69/?view_only=fe23edab18bc40808b1f274b9ceeea15

3
Unsplash.com

4
www.prolific.co

5
www.gorilla.sc
